# Sensitive multiplex PCR assay to differentiate Lyme spirochetes and emerging pathogens *Anaplasma phagocytophilum* and *Babesia microti*

**DOI:** 10.1186/1471-2180-13-295

**Published:** 2013-12-20

**Authors:** Kamfai Chan, Salvatore AE Marras, Nikhat Parveen

**Affiliations:** 1 Department of Microbiology and Molecular Genetics, Rutgers-New Jersey Medical School, 225 Warren Street, Newark, NJ 07103-3535, USA; 2 Public Health Research Institute, 225 Warren Street, Newark, NJ 07103-3535, USA

**Keywords:** *Borrelia burgdorferi*, *Anaplasma phagocytophilum*, *Babesia microti*, Tick-borne emerging pathogens, Real-time PCR, Molecular beacons, Multiplex assay, Lyme disease, Babesiosis, Anaplasmosis

## Abstract

**Background:**

The infection with *Borrelia burgdorferi* can result in acute to chronic Lyme disease. In addition, coinfection with tick-borne pathogens, *Babesia* species and *Anaplasma phagocytophilum* has been increasing in endemic regions of the USA and Europe. The currently used serological diagnostic tests are often difficult to interpret and, moreover, antibodies against the pathogens persist for a long time making it difficult to confirm the cure of the disease. In addition, these tests cannot be used for diagnosis of early disease state before the adaptive immune response is established. Since nucleic acids of the pathogens do not persist after the cure, DNA-based diagnostic tests are becoming highly useful for detecting infectious diseases.

**Results:**

In this study, we describe a real-time multiplex PCR assay to detect the presence of *B. burgdorferi, B. microti and A. phagocytophilum* simultaneously even when they are present in very low copy numbers. Interestingly, this quantitative PCR technique is also able to differentiate all three major Lyme spirochete species, *B. burgdorferi, B. afzelii*, and *B. garinii* by utilizing a post-PCR denaturation profile analysis and a single molecular beacon probe. This could be very useful for diagnosis and discrimination of various Lyme spirochetes in European countries where all three Lyme spirochete species are prevalent. As proof of the principle for patient samples, we detected the presence of low number of Lyme spirochetes spiked in the human blood using our assay. Finally, our multiplex assay can detect all three tick-borne pathogens in a sensitive and specific manner irrespective of the level of each pathogen present in the sample. We anticipate that this novel diagnostic method will be able to simultaneously diagnose early to chronic stages of Lyme disease, babesiosis and anaplasmosis using the patients’ blood samples.

**Conclusion:**

Real-time quantitative PCR using specific primers and molecular beacon probes for the selected amplicon described in this study can detect three tick-borne pathogens simultaneously in an accurate manner.

## Background

*Ixodes* species of ticks are responsible for transmitting Lyme disease causing *Borrelia burgdorferi* and several other pathogens both in the North America and Europe [1,2]. Recently, a press release by Centers for Disease Control and Prevention (CDC) stated that only one tenth (~30,000) of the actual Lyme disease cases, i.e., 300,000, are reported in the United States every year. Several epidemiological studies in these two continents have also shown that in addition to Lyme spirochetes, ticks are often coinfected with the obligate intracellular bacterium, *Anaplasma phagocytophilum*, and a protozoan parasite belonging to the genus, *Babesia* with *B. microti* prevalent in the United States and *B. divergens* in Europe [2-9]. These two are emerging tick-borne pathogens and cause increasing number of infections in the local populations in the endemic zones. *A. phagocytophilum* is the etiological agent of human granulocytic anaplasmosis (HGA) that can manifest as moderate to life-threatening disease in humans. The bacterium preferentially infects granulocytes/neutrophils and persists in polymorphonuclear leukocytes (PMNs), causing thrombocytopenia and leucopenia/lymphopenia, and if untreated, renders the patients susceptible to secondary opportunistic infections. Human babesiosis is an intraerythrocytic infection that may remain asymptomatic but often leads to severe to fatal disease [10]. Sensitive diagnostic tests that can accurately and simultaneously diagnose Lyme disease, anaplasmosis and babesiosis are not currently available emphasizing a need to develop individual test for each pathogen or a combinatorial test for all three tick-borne pathogens to detect coinfection in patients.

*B. burgdorferi, A. phagocytophilum* and *B. microti* have overlapping epidemiology and transmission cycles with shared tick vectors, and common primary and secondary host reservoirs. All three use white-footed mice as a reservoir host and white-tailed deer populations to spread through the endemic regions of the United States [11-14]. HGA and canine granulocytic anaplasmosis, as well as bovine and human babesiosis, are prevalent in Northeastern and Midwestern regions of the United States, as is Lyme disease [8,10,15-23]. Severe to fatal babesiosis cases have been reported in the USA in the past two decades [24,25]. More recently, *A. phagocytophilum* infections have also increased significantly in regions endemic for Lyme disease, with 3,637 HGA cases reported by the CDC in the United States between 2003 and 2008 [26]. The CDC has now declared HGA to be a notifiable disease [26]. In 2002, most commonly diagnosed coinfections in patients in the Eastern parts of the United States were due to *B. burgdorferi* and *B. microti*, accounting for ~80% of the total tick-borne coinfections. These coinfections exhibit more severe clinical symptoms than infections by *B. burgdorferi* and parasite *B. microti* alone probably as a consequence of the modification of the immune system by the latter [20,27]. Coinfections are also prevalent among ticks in Europe and are also becoming common in humans, who are regularly exposed to these ticks [28-30]. Hence, there is a desperate need to develop assays for the detection of pathogens responsible for these diseases individually or together.

Accurate diagnosis of various tick-borne diseases is problematic, due to similar clinical manifestations [12,31]. Currently available serological tests are neither cost-effective, nor sensitive or specific for diagnosis of infections by these three pathogens transmitted by ticks, especially at early stage of infection [9,32-34]. CDC recommends two-tier serological tests with an enzyme-linked immunosorbent assay (ELISA) or indirect immunofluorescence assay (IFA) as primary test followed by the more specific Western blot analysis to confirm diagnosis of Lyme disease [35]. Subjective interpretation of the immunoblots further diminishes accuracy of the test with only 70-80% serological test efficiency noted for diagnosis of Lyme disease. However, accuracy of a single C6 ELISA test sensitivity is reported to be slightly higher for Lyme disease than the two-tier serological test [27]. The positive predictive value of these serological tests depends both on the prevalence of the disease in the area, and on the sensitivity and specificity of the test. Moreover, their predictive value varies among different laboratories depending on which commercial kit is used [36-38]. Furthermore, antibodies persist in the patients long after the disease is cured such that serological tests cannot be used as a test of cure. In addition, it is difficult to assess reinfection in the endemic regions. PCR-based assays have been tried for the diagnosis of Lyme disease, but, by virtue of their design, they have had only a limited level of success [39-41]. *A. phagocytophilum* and *B. microti* infect white and red blood cells, respectively, but are not easily detectable in blood. This offers additional risk since they can also be transmitted through blood transfusions and potentially vertically from mother to infant [19,42-44]. The presence of *Babesia* species is usually visualized by microscopic examination after Giemsa staining; however, it is frequently overlooked, because of the infection of less than 1% of erythrocytes or due to hemolysis during the sample transport. Higher parasitemia due to Babesia infection is usually fatal. Serological tests and PCR have been found to be more sensitive for its detection [45,46]. Microscopic detection of *A. phagocytophilum* morulae in blood smears is also difficult because <0.1% of neutrophils may show their presence [47]. Like *B. burgdorferi*, *A. phagocytophilum* lacks lipopolysaccharides and displays a large number of immunogenic proteins on the bacterial surface, making serological tests feasible. However, similar to Lyme disease, serodiagnosis of HGA fails to detect active disease [34,48,49]. Therefore, an assay that can identify these two tick-borne pathogens, in addition to detecting Lyme spirochetes will be ideal, cost-effective and will facilitate design of proper treatment strategies for bacteria versus parasite.

Due to the presence of nucleases in the serum, nucleic acids of the pathogens do not persist in the host much longer after the disease is cured [50]. Therefore, PCR and other nucleic acids-based assays have been used as test of cure for a variety of infectious diseases [51-53]. Selection of proper PCR targets and conditions along with the use of efficient detection probes are critical for the development of sensitive and specific diagnostic assays. Molecular beacons are hairpin-shaped oligonucleotide probes that are highly specific for their target sequences and can be labeled with distinguishably colored fluorophores [54]. The single-stranded loop of molecular beacons is designed to be complementary to a unique gene sequence that identifies the infectious agent. Drs. Marras, Tyagi, and Kramer used these probes to distinguish alleles that differ in as little as a single nucleotide polymorphism (SNPs) [55,56]. The basis of this extraordinary specificity is that hairpin-shaped probes can assume two different stable states, by: (i) forming double-stranded hybrids with their target sequence, or (ii) retaining their partially double-stranded structure when not bound to a target. Any mismatch between the probe sequence of the molecular beacon and the target sequence destabilizes the probe-target hybrid, leading to return of the molecular beacon in its stable hairpin structure [57,58]. Unlike hairpin-shaped probes, linear probes such a TaqMan probes have only one conformation, either on or off the target. This decreases difference between the melting temperature of a perfectly matched target sequence and a single-nucleotide mismatched target sequence makes discrimination between two scenarios more difficult to discern [58-60]. Furthermore, Taqman probes are digested by the endonuclease activity of the Taq polymerase in each PCR cycle, such that optimization of both annealing and digestion of the probe becomes more challenging in the development of multiplex assays. Our success in utilizing the extraordinary specificity of molecular beacon probes to detect the *recA* gene of *B. burgdorferi*, and to quantitate the number of spirochetes present in infected mouse tissue [61] offered us an incentive to develop the assay for diagnosis of Lyme disease in humans. We have now optimized the assay to work in the presence of human DNA for it to become useful as diagnostic test for human Lyme disease. We describe here expansion of a simplified, highly sensitive multiplex real-time PCR assay by incorporating specific molecular beacons that can distinguish *B. burgdorferi*, *A. phagocytophilum* and *B. microti* simultaneously. Application of this assay will make a significant difference in achieving the rapid and accurate diagnosis of Lyme disease, anaplasmosis and babesiosis in a cost-effective manner.

## Methods

### Microbial strains and human cell line

For standardization of conditions for real-time PCR diagnostic assay for Lyme disease, N40 strain clone D10/E9 of *B. burgdorferi* (sensu stricto), VS461 strain of *B. afzelii* and PBi strain of *B. garinii* were grown in BSKII medium supplemented with 6% rabbit serum at 33°C. Dr. Edouard Vannier of Tufts Medical Center at Boston, and Dr. Errol Fikrig of Yale University School of Medicine generously provided the genomic DNA from *B. microti* strain RM/NS and *A. phagocytophilum* strain HZ, respectively. Human embryonic kidney 293 cells were cultured in a 1:1 mix of DMEM (low glucose) and Ham’s F12 medium (Life Technologies, NY) supplemented with 10% FBS to isolate human DNA used in the assays.

### Isolation of *B. burgdorferi* and human genomic DNA

Total genomic DNA was isolated from the Lyme spirochetes grown to a density of ~10^8^ spirochetes/ml and from 293 cells using the previously described protocols [62,63] with two modifications. First, PLG-containing tubes (Qiagen Sciences, MD) were used for phenol and chloroform extraction, since they allow clean separation of the top aqueous layer by decantation after centrifugation. Second, a final step of passing the DNA through DNeasy kit columns (Qiagen Sciences, MD) was included to obtain good quality DNA for real-time PCR.

### *B. microti* and *A. phagocytophilum* plasmid construction

Thiamine pyrophosphokinase gene of *B. microti* (BmTPK) and APH1387 gene of *A. phagocytophilum* were amplified from *B. microti* strain RM/NS and *A. phagocytophilum* strain HZ, respectively, using primers listed in Table 1, which are designed specifically for RM/NS and HZ strains genes, respectively. Each PCR amplicon was cloned in pCR-XL-TOPO vector (Life Technologies, NY). Plasmid containing BmTPK or APH1387 gene was used as template in real-time PCR assays.

**Table 1:** Sequence of PCR primers and molecular beacon probes

**PCR primers/Probes/Oligos**	Sequence*	**Length**	**Tm (°C)**	**Size of PCR amplicon**	**Fluorophore/Quencher**
RecF primer	5’ GTG GAT CTA TTG TAT TAG ATG AGG CTC TCG 3’	30	66.1	222 bp	
RecR primer	5’ GCC AAA GTT CTG CAA CAT TAA CAC CTA AAG 3’	30	67.3		
RecF3 primer	5’ GCA AGA GTT CAA ATA GAA AA 3’	20	53.7	287 bp	
RecR3 primer	5’ AAA GCT TTT GCA TAA ACA G 3’	19	54.7		
RecA3 probe	5’ *CTG* ***GCG *** **GAT ATC CTA GGG GG *CGC*** *CAG* 3’	26	67.9		FAM/ BHQ-1
5BmicrotiTPK primer	5’ AAT ATT GTT GAA TGG GGA TAT TTG TG 3’	26	64.2	600 bp	
3BmicrotiTPK primer	5’ AAT AAT ATA GCT TTT CCA AAA TAT AAC TGA C 3’	31	60.2		
5BmTPK primer	5’ TGA GAG GAA CGA CCA TAG C 3’	19	61.4	141 bp	
3BmTPK primer	5’ CCA TCA GGT AAA TCA CAC GAA A 3’	22	61.6		
BmTPK probe	5’ *CGC GTC***GGT GTT GTT GAC CAG CGG CCG CG***GAC GCG* 3’	35	61.5		CAL Fluor Orange 560/ BHQ-1
5ApAPH1387 primer	5’ ATG TAT GGT ATA GAT ATA GAG CTA AGT GA 3’	29	57.8	1737 bp	
3ApAPH1387 primer	5’ CTA ATA ACT TAG AAC ATC TTC ATC GTC AG 3’	29	62.2		
5Aphagocyt primer	5’ ATG GCT ACT ACG AAG GAT 3’	18	57.9	152 bp	
3Aphagocyt primer	5’ CGA AGC AAC ATC TCT ACA T 3’	19	58.0		
Aph1387 probe	5’ *CGG TGC***GAC AAA GAT GCC AGC ACT AAT GCG***GCA CCG* 3’	36	61.9		CAL Fluor Red 610/ BHQ-2
5ACTA1 primer	5’ AGA GCA AGA GAG GTA TCC 3’	18	58.0	104 bp	
3ACTA1 primer	5’ CTC GTT GTA GAA GGT GTG 3’	18	57.7		
ACTA1 probe	*5’ CGC TGC***CCT ATC GAG CAC GGC ATC ATC AC***GCA GCG* 3’	35	62.4		Quasar 670/ BHQ-2
RecA3MB-com oligo	5’ ttG CGC CCC CTA GGA TAT CCG Ctt 3’	24	67.9		
TPKMB-com oligo	5’ tt tCG CGG CCG CTG GTC AAC AAC ACC ttt 3’	29	61.5		
AphMB-com oligo	5’ ttt CGC ATT AGT GCT GGC ATC TTT GTC ttt 3’	30	61.9		
ActinMB-com oligo	5’ tt tGT GAT GAT GCC GTG CTC GAT AGG ttt 3’	29	62.4		

*Italicized molecular beacon sequence depicts the arm sequences whereas the sequences marked by bold letters indicate probe region of molecular beacons complementary to the target sequence.

### Isolation of total DNA from blood and blood culture

To determine the sensitivity of detection of spirochetes in blood, we inoculated ten-fold diluted spirochetes starting from 10^4^ in 1.5 ml human whole blood. Duplicate sets of three replicates for each dilution were prepared. Total DNA from one set of tubes was isolated immediately while 1.5 ml BSKII medium with 6% rabbit serum was added to the second set of tubes. Total DNA from this set of tubes was isolated using the method described above after incubation of the tubes at 33°C for 48 h. From 100 μl of total DNA suspension, 5 μl of sample was used for real-time PCR. Unspun human whole blood with EDTA was purchased from Biological Specialty Corporation (Colmar, PA) through Fisher Scientific. Experiment with the human blood was conducted under the protocol of the corresponding author approved by the Institutional Review Board of New Jersey Medical School. DHHS Federal Wide Assurance is provided to New Jersey Medical School for work involving human samples. Since no patients participated in this study, consent form was not needed.

### Molecular beacon design

Design of molecular beacon probe to hybridize to the *recA* gene of Lyme spirochetes and tagged with FAM fluorophore and BHQ-1 quencher were described previously [61]. Other molecular beacon probes were designed using the previously described strategies [64]. Briefly, molecular beacon probes for; ACTA1 gene amplicon was tagged with Quasar 670 fluorophore and BHQ-2 quencher, BmTPK amplicon with CAL Fluor Orange 560 fluorophore and BHQ-1 quencher and APH1387 amplicon using CAL Fluor Red 610 and BHQ-2 quencher. The lengths of the probe sequences were chosen so that they would form a stable hybrid with the target at the annealing temperature (60°C) of the PCR assay. The 5’ and 3’ arm sequences of the molecular beacons were designed to form a stable hybrid at 5 to 10°C above the annealing temperature of the PCR assay. The fluorophores and quenchers were chosen based on the specifications of the spectrofluorometric thermal cycler platform on which the assays were carried out and their compatibility in one multiplex assay. The sequences of the molecular beacons used in this study are listed in Table 1. A detailed protocol for the synthesis and purification of molecular beacons can be found at http://www.molecular-beacons.org. For this study, molecular beacons were ordered from Biosearch Technologies, CA. Initial standardization of PCR conditions was conducted by using SYBR Green I dye (Life Technologies, NY) and was followed by replacing SYBR Green with specific molecular beacon probes in the assays.

### Real-time PCR assays

Since genome sizes of *B. burgdorferi* and human are 1.5 Mb and 3.2 Gb respectively, 2 ng of *B. burgdorferi* genomic DNA contains approximately 10^6^ copies of *recA* gene, while 350 ng of human genomic DNA contains approximately 10^5^ copies of ACTA1 gene. A 222 bp fragment from *recA* gene of *B. burgdorferi* using RecF and RecR primers and a 104 bp fragment from human alpha actin A1 (ACTA1) gene using 5ACTA1 and 3ACTA1 primers were amplified by PCR in 0.2 ml optical tubes using a Bio-Rad CFX96 Touch Real-time PCR system (Bio-Rad Life Science Research, CA). Amplification was performed in 25 μl reaction mixtures containing AmpliTaq Gold PCR reaction buffer (Life Technologies, NY) supplemented with 3 mM MgCl_2_, 500 ng/μl of bovine serum albumin, 250 μM of each deoxynucleoside triphosphate (dNTP), 500 nM of each set of primers, 5 units of AmpliTaq Gold polymerase (Life Technologies, NY), and 100 nM each of RecA3 and ACTA1 molecular beacon probe. Specificity of each primer set and molecular beacon probe was first checked in monoplex assays using the specific primers/probe in the PCR. The primer/probe sets of other pathogen(s) were included as negative controls in these assay (data not shown). For each amplification reaction, 5 μl of the DNA template was used to minimize the variation due to pipetting error. The amplification program consisted of initial heating at 95°C for 5 minutes, followed by 50 cycles of heating at 95°C for 15 s, annealing and fluorescence detection at 60°C for 30 s, and polymerization at 72°C for 20 s. Similarly, amplification of a 141 bp amplicon from BmTPK gene using 5BmTPK and 3BmTPK primers and a 152 bp amplicon of APH1387 gene using 5Aphagocyt and 3Aphagocyt primers were carried out in the presence of human genomic DNA. Molecular beacon probes, BmTPK and APH1387 were used for detection of the respective amplicons. All primer and probe sequences are listed in Table 1. Data were processed using the software provided by the manufacturer.

### Quadruplex real-time PCR assays

Quadruplex real-time PCR assay was performed in conditions described above. Genomic DNA of *B. burgdorferi* and human, and clones of BmTPK and APH1387 were used as templates, and 500 nM each of RecF and RecR primers and 5BmTPK and 3BmTPK primers, 250 nM each of 5Aphagocyt and 3Aphagocyt primers, 100 nM each of 5ACTA1 and 3ACTA1 primers, 100 nM each of RecA3, BmTPK, APH1387, and ACTA1 molecular beacons were included in each reaction.

For confirmation of the quadruplex assay in which plasmids containing BmTPK and APH1387 were used, we incorporated different concentrations of genomic DNA of *B. burgdorferi*, *B. microti* and *A. phagocytophilum* in the triplex real-time PCR. Human DNA control was not included in these assays. Genome sizes of *B. microti* and *A. phagocytophilum* are 6.5 Mb and 1.47 Mb, respectively. Therefore, 10^6^ copies of BmTPK and APH1387 are calculated to be present in 8 ng and 2 ng of genomic DNA, respectively. By using different relative genomic copy numbers and the conditions described above for quadruplex assay, consistent results validated our assay for simultaneous detection of all three pathogens.

### Borrelia speciation by real-time PCR assays

To differentiate three major species that cause Lyme disease in Europe, *B. burgdorferi*, *B. afzelii* and *B. garinii*, asymmetric PCR assay was performed in 25 μl volume such that the primer synthesizing the target strand of the molecular beacon was used in excess. The primers for *recA* gene that are from the conserved region in all three species, RecF3 and RecR3 were designed to amplify a slightly longer 287 bp fragment in this asymmetric PCR assay. The reaction mixture contained AmpliTaq Gold PCR buffer supplemented with 3 mM of MgCl_2_, 500 ng/μl of bovine serum albumin, 250 μM of each dNTP, 30 nM of RecF3 primer, 1000 nM of RecR3 primer, 50 nM of RecA3 molecular beacon and 5 units of AmpliTaq Gold polymerase. The amplification program consisted of initial heating at 95°C for 5 minutes, followed by 60 cycles of heating at 95°C for 15 s, annealing and fluorescence detection at 60°C for 30 s, and polymerization at 72°C for 20 s. It was immediately followed by incubation at 25°C for 2 minutes to allow annealing, and then a melt curve was included by increasing the temperature from 25°C to 95°C in 1°C step, with each step lasting 2 minutes while monitoring the fluorescence. For analysis, the first derivative of the denaturation profile was determined as described previously [51].

## Results

### Optimization of molecular beacon probes for multiplex PCR assays

**Figure 1:**
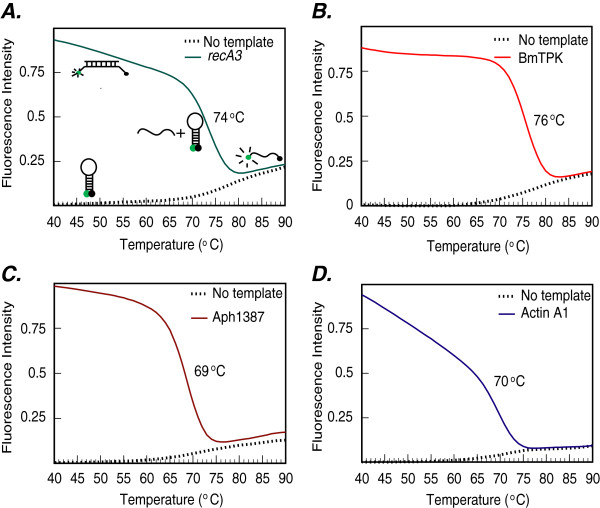
**Denaturation profile analysis of molecular beacon probes used in this study.** Melting curves of the RecA3 molecular beacon **(A)** in the presence of a complementary target sequence (green line), or in the absence of any target (buffer only control, dotted line) were generated. The fluorescence intensities indicate that the molecular beacon exists either as a hybrid with its perfect complementary target sequence, exhibiting high fluorescence from 25°C to 55°C, or in its free state in the form of a stem-loop structure with fluorescence quenched in a temperature range of 25–65^o^C as depicted by the cartoons. At higher temperatures (more than 70^o^C) the molecular beacon probe denatures and exhibits high fluorescence intensities in control. Similarly, probe-target hybrid also denatures at higher temperature releasing the target and diminishing the fluorescence as the probe returns to hairpin-loop structure. A similar analysis of the BmTPK, APH1387 and ACTA1 molecular beacon probes depicted a temperature and fluorescence profile **(B, C, and D)**, which is similar to the RecA3 molecular beacon probe.

To develop and optimize the multiplex assay that can detect the presence of three tick-borne pathogens along with the human DNA control in the patient sample, we selected primers and molecular beacon probes that will amplify and detect the amplicons under the same selected PCR parameters. The absence of amplification of the amplicons of each pathogen in the presence of primers of other pathogens confirmed the specificity of each set of primers for only the relevant pathogen template DNA. The specificity of each molecular beacon for its respective amplicon was first determined by generating the denaturation profiles for each probe in the absence or presence of specific oligonucleotides (Figure 1 and Table 1). In the presence of the unrelated target or in the absence of any target (buffer control), RecA3, BmTPK, APH1387 and ACTA1 molecular beacons remain in a closed state at low temperatures with fluorophore and quencher held in close proximity by the hairpin formation (Figure 1A). Molecular beacons remain dark at this state. At temperature above the melting temperatures of the stems (~68°C, 62°C, 62°C and 63°C for RecA3, BmTPK, APH1387, and ACTA1, respectively), the fluorophore separates from the quencher resulting in increase in fluorescence intensity. The molecular beacons bind to their respective targets at low temperature resulting in the dissociation of the stem and a high level of fluorescence. In contrast, at the melting temperatures of probe-target hybrids (74°C, 76°C, 69°C and 70°C for RecA3, BmTPK, APH1387, and ACTA1, respectively), dissociation of the probe from the target results in the return of the probe to a stem-loop structure, significantly diminishing the fluorescence. On further increase in temperature, the beacons denature completely, do not form a stem-loop structure, and hence, start to emit some fluorescence (Figure 1A to 1D). Cartoons in the Figure 1A depict different molecular beacon states at particular temperatures, in the presence or absence of specific targets in the reaction.

### Detection of *recA* amplicon of *B. burgdorferi* in the presence of human genomic DNA in a multiplex real-time PCR assay

**Figure 2:**
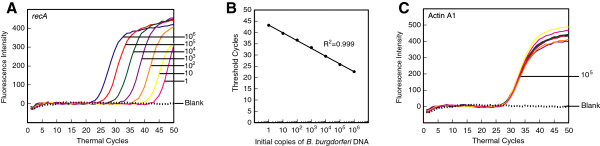
**Molecular beacons can detect *B. burgdorferi *between 1 and 10**^**6 **^**in a duplex assay, when human DNA was also included.** Amplification plots of *recA* and Actin A1 genes in PCR assays to estimate quantities of *B. burgdorferi***(A)** and human **(C)** DNA are shown. Human DNA (containing 10^5^ Actin A1 gene copies) spiked with ten-fold dilutions of *B. burgdorferi* strain N40 ranging from 1 to 10^6^ were used in the PCR assays containing both RecA3 and ACTA1 molecular beacons. Sensitivity and specificity of the detection system is indicated by the ability of RecA3 and ACTA1 molecular beacons to quantitatively detect the amplicons from both the *recA* and the ACTA1 genes in the same PCR assay tubes. A high coefficient of correlation (r^2^ = 0.999) between the Ct values and the spirochete number obtained from the standard curve **(B)** indicates that the molecular beacons can be used effectively to quantify spirochete burden in infected tissues using multiplex assay system.

We had already optimized molecular beacons and PCR conditions for quantitative detection of *B. burgdorferi* DNA by real-time PCR [61]. To adapt the assay for diagnosis of Lyme disease in the patients, we spiked the same quantity of human DNA (350 ng genomic DNA or 10^5^ ACTA1 copy number) with a ten-fold dilution of genomic DNA of *B. burgdorferi*. Since simultaneous detection of pathogen and host PCR products is possible when molecular beacon probes are tagged with different fluorophores, normalization of the host DNA in patient sample will be convenient and accurate method to quantify spirochete number, if needed. In addition, accurate detection of host DNA in each sample will ensure that the quality of the isolated DNA was suitable for real-time PCR. To evaluate this premise, we included primers and molecular beacons for both *recA* amplicon of *B. burgdorferi* and ACTA1 amplicon of human DNA. Amplification plots of the *recA* gene in the PCR assays (Figure 2A), as detected by fluorescence intensity at the end of each cycle at the annealing temperature, show that the presence of 1 to 10^6^ spirochetes can be detected using the RecA3 molecular beacon consistently. A standard curve (Figure 2B) generated by plotting the threshold cycle (Ct) versus the log of the known initial copy numbers of *B. burgdorferi* indicates that the threshold cycle is inversely proportional to the number of target molecules present in the samples. A high coefficient of correlation (r^2^ = 0.999) between the *B. burgdorferi* copy number and the Ct obtained from the standard curve indicates that this curve can accurately determine the quantity of spirochetes in infected patient samples. Results obtained in monoplex and multiplex assays did not show significant differences (data not shown). In addition, identical Ct values for ACTA1 in all samples were detected, indicating that variation in the copy number of *B. burgdorferi* genome, or the presence of the human DNA in the sample does not affect sensitivity of detection of amplicons of the pathogen or the host in the multiplex assay (Figure 2A, 2C and data not shown).

### TPK gene amplicon of *B. microti* can be detected efficiently along with human ACTA1 in a multiplex PCR assay

**Figure 3:**
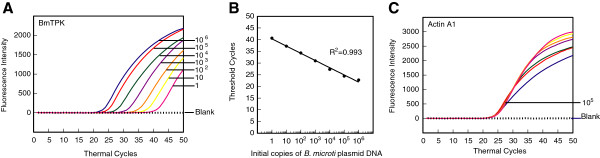
**Molecular beacons can detect DNA between 1 and 10**^**6 **^***B. microti *in a duplex assay in the presence of human DNA.** Amplification plots of BmTPK and ACTA1 genes in PCR assays using the human DNA representing 10^5^ ACTA1 copies spiked with ten-fold dilutions from 1 to 10^6^ of *B. microti* DNA copies were used to estimate quantities of *B. microti***(A)** and human **(C)** DNA by employing both BmTPK and ACTA1 molecular beacons. The assay quantified amplicons from both the BmTPK and the ACTA1 genes in the same PCR assay tubes. A high coefficient of correlation (r^2^ = 0.993) between the Ct values and the parasite numbers obtained from the standard curve **(B)** indicates that the molecular beacons can be used effectively to quantify the parasite burden in the infected human cells using multiplex assay system using the optimized conditions.

Two enzymes were identified to be important in central metabolism of *B. microti* by genome sequencing of this parasite [65], Lactate dehydrogenase (LDH) and TPK. Only LDH is expressed during intra-erythrocytic multiplication stage of this pathogen. We cloned both LDH and TPK genes and initially used both plasmid clones as templates for real-time PCR using SYBR green and also respective molecular beacons (data not shown). However, only BmTPK showed promising results under conditions optimized for amplification of Lyme spirochetes and *A. phagocytophilum* gene amplicons. Therefore, we conducted further investigation using the BmTPK gene only. Ten-fold dilutions of plasmid containing BmTPK gene, starting with 10^6^ copies, were prepared in the human DNA suspension (350 ng) containing 10^5^ copies of ACTA1 to use as template. Using 5BmTPK and 3BmTPK primers, BmTPK molecular beacon in addition to human actin A1 primers and probe and following the PCR conditions described in the methods section, amplification of TPK and ACTA1 amplicons were detected and quantified. Although copy number from 10^6^ to 10 of BmTPK showed consistent results (Figure 3A), detection of single copy number of *B. microti* DNA was slightly less reproducible. Standard curve (Figure 3B) depicts the precision of these results with significant coefficient of correlation (r^2^ = 0.993). Thus, it is expected that 10 copies of TPK will be detected consistently in this assay with lower detection limit often possible. Overlapping ACTA1 detection curves indicate the accurate detection and quantitation of the human amplicon since the same concentration of human DNA was used in different tubes for dilution of TPK-containing plasmid (Figure 3C).

### Specific detection of APH1387 amplicon in the presence of human DNA using molecular beacon probes in a multiplex PCR assay

**Figure 4:**
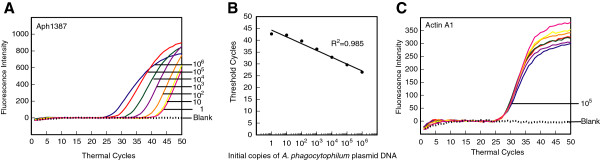
**Molecular beacons can detect DNA between 1 and 10**^**6 **^***A. phagocytophilum *in a duplex assay when the human DNA is also present.** Amplification plots of APH1387 and ACTA1 genes in PCR assays using the human DNA representing 10^5^ ACTA1 copies spiked with ten-fold dilutions from 1 to 10^6^ plasmid copies containing APH1387 were used to estimate quantities of *A. phagocytophilum***(A)** and human **(C)** DNA by employing both Aph1387 and ACTA1 molecular beacons. The assay quantified amplicons from both the APH1387 and the ACTA1 genes in the same PCR assay tubes. A high coefficient of correlation (r^2^ = 0.985) between the Ct values and the bacterial numbers obtained from the standard curve **(B)** indicates that the molecular beacons can quantify burden of this intracellular pathogen in the infected human cells using multiplex assay system under the standardized conditions in a sensitive and specific manner even though sensitivity of detection is slightly higher than one.

*A. phagocytophilum* is an obligate intracellular pathogen that multiplies within a vacuole inside the host cells and avoids fusion of this vacuole with lysosome. APH1387 of *A. phagocytophilum* was identified as the first protein that localizes to the vacuolar membrane containing this pathogen [66]. Since the gene is uniquely present in *A. phagocytophilum* and is highly conserved in various strains, it will allow detection of this pathogen in patient samples irrespective of the presence of different infecting strains. Therefore, we selected this amplicon for detection of this bacterial pathogen by real-time PCR. By using the strategy used for TPK gene containing plasmid for *B. microti* described above, APH1387 containing plasmid was diluted in human DNA and PCR was conducted using 5Aphagocyt and 3Aphagocyt primers and Aph1387 molecular beacon. Primers for human actin A1 gene amplicon and ACTA1 molecular beacon were also included in the reaction mixture. Conditions for PCR were identical to those used for Lyme spirochetes *recA* and *B. microti* TPK gene amplifications. Interestingly, in repeated experiments, APH1387 detection limit was similar to that of BmTPK (Figure 4A) and sensitivity of detection appears to be slightly lower (>1 bacterial amplicon) than the detection limit for *recA* amplicon of Lyme spirochetes (~1). Indeed, the curves for 10 and 1 copies of the gene were very close to each other. Again, the results were reflected in the standard curve and slightly lower coefficient of correlation (r^2^ = 0.985) (Figure 4B) than that for *recA* (r^2^ = 0.999). Sensitivity of detection of human ACTA1 amplicon was maintained (Figure 4C) similar to that in the multiplex assays described for *recA* and BmTPK amplicons above.

### Simultaneous detection of *recA* of Lyme spirochetes, TPK of *B. microti* and APH1387 amplicon of *A. phagocytophilum* along with human actin A1 in a quadruplex PCR assay

**Figure 5:**
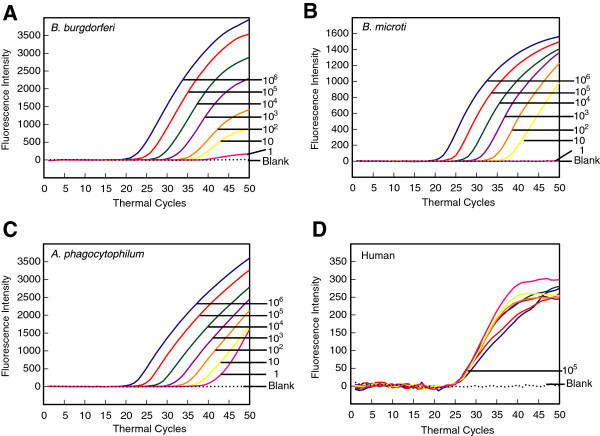
**Inclusion of three tick-borne pathogens in the presence of human DNA in a single quadruplex assay does not affect the sensitivity of their detection.** Conditions for a quadruplex PCR assay were optimized such that eight primers and four different molecular beacons for respective amplicons were present in the same tube along with the other reagents required for the PCR. Sensitivity of detection of two bacterial pathogens, extracellular spirochete *B. burgdorferi***(A)** and obligate intracellular pathogen *A. phagocytophilum***(C)***,* along with the intracellular parasite, *B. microti***(B)**, was not affected in this quadruplex assay, indicating that the assay can be extended for simultaneous diagnosis of all three tick-borne pathogens in the patients, especially in the endemic regions. Detection of the ACTA1 amplicon in the same reaction will offer as control for human DNA **(D)** and quality of DNA preparation when the patient samples will be used for diagnosis of the infecting organism.

Since coinfection of ticks with Lyme disease spirochetes and emerging pathogens *Babesia* species and *A phagocytophilum* has been increasing in the endemic regions of tick-borne illnesses, it is very likely that these coinfections will continue increasing steadily in humans in the near future. Therefore, development of a single multiplex real-time PCR assay for simultaneous detection of all three tick-borne pathogens in the patient samples in a sensitive and specific manner is expediently warranted. Even though cloned genes of both pathogens, *B. microti* and *A. phagocytophilum*, in plasmids could be detected and quantitated when present individually, it is essential to determine if the sensitivity is maintained when the DNA of all three pathogens is present in the assay. To achieve this goal, we standardized conditions such that genomic DNA of *B. burgdorferi* and plasmids containing BmTPK and APH1387 genes were serially diluted in human DNA containing 10^5^ copies of ACTA1 gene. Sensitivity of detection of *recA* amplicon was not affected by the presence of DNA of other two pathogens (Figure 5A). By increasing the concentration of molecular beacons in the quadruplex assay mixture, we were able to improve the sensitivity of detection of *A. phagocytophilum* APH1387 amplicons such that one copy number was clearly distinguishable from 10 DNA copies (Figure 5B). However, based upon Poisson distribution, an average single copy of the template is not expected to be present in all samples consistently. Lack of amplification of predicted one copy of *B. microti* in this assay demonstrates this probability (Figure 5C). This assay demonstrated that amplicons from all three pathogens along with the control human ACTA1 gene amplicon can be detected accurately in one multiplex assay (Figure 5D) and sensitivity of detection of different pathogens was not affected. This is a highly promising result that will lead to expansion of this assay to the patient samples from endemic regions in the future.

### Sensitivity of detection of emerging pathogens *B. microti* and *A. phagocytophilum* DNA is retained in the presence of excess of *B. burgdorferi* DNA

**Figure 6:**
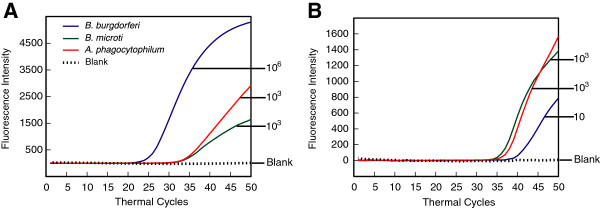
**Sensitivity of detection of tick-borne pathogens *B. burgdorferi, B. microti*, and *A. phagocytophilum *are not affected in the presence of excess of other pathogens. (A)** One thousand copies of *B. microti* and *A. phagocytophilum* genomic DNA were accurately detected in the triplex assay despite 10^3^-fold excess of copy number of *B. burgdorferi* genomic DNA. **(B)** Detection of ten *B. burgdorferi recA* amplicon copies was not affected in the triplex assay even in the presence of 100-fold excess of copy number of both *B. microti* and *A. phagocytophilum* genomic DNA.

Depending on the prevalent conditions in a particular endemic region, quantities of these emerging pathogens may vary in the patient samples. Therefore, we further assessed the sensitivity of the assay for detection of *B. microti* and *A. phagocytophilum* in excess of *B. burgdorferi* DNA. We used *B. burgdorferi* genomic DNA/*recA* copy number (10^6^) along with genomic DNA equivalent to 10^3^ genomic copies of each of *B. microti* and *A. phagocytophilum* (Figure 6A). Accuracy and sensitivity of detection of *B. microti* and *A. phagocytophilum* was not affected by 10^3^-fold excess of *B. burgdorferi* genomic DNA, validating the potential of our multiplex assay for diagnosis of all three tick-borne infections even if one pathogen is present in excess. Such excess of *B. burgdorferi* may be present in the synovial fluid or skin biopsy samples from the patients.

### *B. burgdorferi* can be accurately detected even in the 100-fold excess of *B. microti* and *A. phagocytophilum* genomic DNA

Blood is primarily used as conduit by Lyme spirochetes to disseminate to various tissues such that usually only a few *B. burgdorferi* are present in the blood at any given time. Therefore, it is likely that intracellular blood-borne pathogens *A. phagocytophilum* and *B. microti* could be present in higher numbers in the cells even if the patient has coinfection with *B. burgdorferi*. To determine whether detection of *B. burgdorferi* will be affected by the presence of higher levels of bacteremia and parasitemia due to *A. phagocytophilum* and *B. microti*, respectively, we mixed genomic DNA of all three pathogens such that the copy number of BmTPK and APH1387 was 100-fold higher than that of the *recA* copies of *B. burgdorferi*. Interestingly, we were able to consistently detect ten copies of *recA* per one thousand copies of BmTPK and APH1387 in a multiplex assay (Figure 6B). These results in the Figure 6 demonstrate that irrespective of the levels of each pathogen quantity relative to the other two pathogens, our multiplex assay can accurately detect and even quantify each pathogen in the mixture.

### Differentiation of Lyme spirochetes using denaturation curve analysis

**Figure 7:**
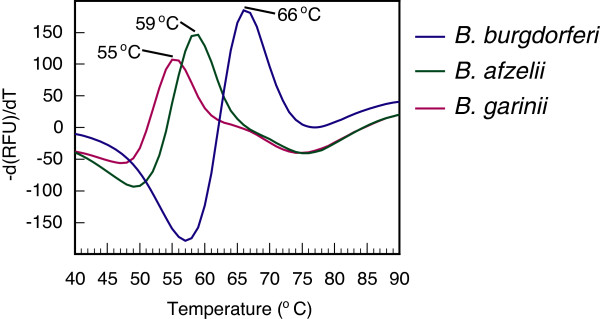
**Denaturation profiles can distinguish three major Lyme spirochete species.** Amplification of 287 bp amplicons from *B. burgdorferi, B. afzelii* and *B. garinii* by real-time PCR using conserved primers was followed by a denaturation profile analysis. SNPs in the molecular beacon-binding region of *B. burgdorferi, B. afzelii* and *B. garinii* resulted in at least 4°C melting temperature difference between the species such that RecA3 molecular beacon was able to distinguish all three *Borrelia* species when first derivative analysis of the denaturation profile was conducted.

The PCR assay for *B. burgdorferi* described in Figure 2 failed to both amplify and detect *B. afzelii* and *B. garinii* amplicons efficiently and differentiate these three Lyme spirochetes. Inefficiency of the PCR amplification for *B. afzelii* and *B. garinii* amplicons is likely due to the presence of SNPs found in the RecF and RecR primers binding sites in these two species. RecF and RecR primers were designed based upon *B. burgdorferi* sequence. Therefore, conserved primers RecF3 and RecR3 were selected for amplification of a 287 bp size amplicon of the *recA* gene by PCR all three species. These primers amplified the gene fragment from all three species efficiently. To clearly distinguish three *Borrelia* species using the denaturation profiles, we conducted asymmetric PCR in which RecR3 primer that synthesizes DNA strand targeted by molecular beacon probe was used in excess. This significantly increases the availability of amplified DNA target for the RecA3 probe to bind. SNPs that are present in the probe-binding region of the amplicon affect the temperature required to denature the probe-target hybrid. Indeed, denaturation profile obtained after asymmetric PCR completion was able to distinguish three *Borrelia* species, with a melt peak of 66°C for *B. burgdorferi*, 59°C for *B. afzelii,* and 55°C for *B. garinii* (Figure 7).

### Real-time PCR can successfully detect low numbers of *B. burgdorferi* in human blood

**Figure 8:**
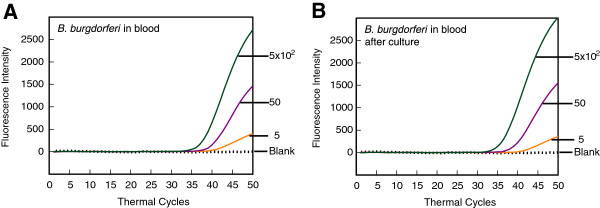
**Multiplex assay using 1.5 ml human blood spiked with serial dilutions of Lyme spirochetes can recover and quantitate *B. burgdorferi*. (A)***B. burgdorferi* were detected consistently in all replicates when ≥5 bacteria were present per ~75 μl of blood, i.e., when 5 μl of total 100 μl DNA recovered from 1.5 ml spiked blood was isolated without additional manipulation. Detection of human Actin A1 was not affected in the multiplex assay, as expected (data not shown). **(B)** Improvement in recovery and quantitation of *B. burgdorferi* after 48 h culture of Lyme spirochetes spiked human blood in BSKII + RS medium at 33°C was not significant.

In this final assay, we determined if the presence of spirochetes could be detected in the human blood. DNA isolated from blood spiked with live spirochetes, with or without culture in BSKII + RS medium, was used as template for real-time PCR for *recA* amplicon of *B. burgdorferi* (Figure 8A and 8B). Detection of spirochete DNA did not significantly improve after culture when the number was close to 1 per 1.5 ml of blood. The presence of 10 spirochetes in 1.5 ml of blood could be consistently detected albeit without accurate quantification irrespective of blood culture (data not shown). Quantitation of 100 spirochetes in 1.5 ml of blood or 100 μl of total DNA isolated from spiked blood (i.e. 5 spirochetes per 5 μl of template used in PCR) was accurate and consistent both with and without culture in BSKII + RS. Thus, the sensitivity of detection in this assay remains better than in any other nucleic acids based assays for Lyme spirochetes described previously.

## Discussion

Lyme disease is prevalent in both the Unites States and Europe. Although *B. burgdorferi* sensu stricto is documented to be the spirochete responsible for Lyme disease in the USA, *B. afzelii* and *B. garinii* affect a significant population in Europe and Asian countries [67,68]. Emerging pathogenic disease anaplasmosis caused by *A. phagocytophilum* is one of the most prevalent life-threatening tick-borne diseases and has recently become notifiable in the United States [14,69]. Furthermore, *B. microti* in the USA and *B. divergens* in Europe have become important tick-borne parasitic diseases and infections with these pathogens are increasing steadily [10,70]. Another major upcoming problem is blood transfusion associated babesiosis that can remain undetected and result in fatalities, and thus, is becoming a blood safety threat [71-74]. Serological tests used for diagnosis of Lyme disease, anaplasmosis and babesiosis cannot be used early in infection before the adaptive immune response is established. In addition, due to persisting antibodies long after disease has resolved and patient is cured, these tests cannot be used to detect active infection and they fail as test of cure. These difficulties add to the disadvantage of using the indirect serological diagnostic tests for tick-borne infectious diseases. Furthermore, species and strains differences in different geographical regions could further diminish the efficient diagnosis with the available commercial serological kits [37]. In addition, microscopic examination for diagnosis of anaplasmosis and babesiosis is both time-consuming and labor intensive making them quite expensive. Hence, there is a desperate need to develop efficient tests for detection of the presence of these pathogens in a cost-effective and efficient manner.

The presence of nucleases in serum and in other body fluids ensures clearance of nucleic acids when pathogens are eliminated by treatment with antimicrobials [50,75,76]. Therefore, nucleic acid based tests are now becoming popular for diagnosis of various infectious diseases [51,52,77]. Indeed, these assays are ideal as the tests of cure for various diseases. Early detection of infection by *Borrelia* species, *A. phagocytophilum* and *Babesia* species using nucleic acid based techniques can lead to successful treatment of the illnesses in a timely manner. We previously developed a sensitive and accurate quantitative real-time PCR assay using molecular beacons for mouse tissues [61]. MassTag PCR has been employed to detect coinfection of ticks collected from different sites in New York with *B. burgdorferi*, *A. phagocytophilum* and *B. microti*[6,78] and quantitative PCR has also been employed recently for patient samples [79]. A pilot study, using the patient blood samples used multi-locus PCR and electrospray ionization mass spectrometry, showed 90% efficiency in detection of early Lyme disease and could often distinguish different strains/genotypes involved [80]. Recently, a real-time PCR test using 18S rRNA gene of *B. microti* was successfully used by employing small DNA groove probe for specific detection of the presence of this parasite with a sensitivity of ~100 gene copies per 5 μl of the patients’ blood [53]. However, all these tests have yet to be fully refined to employ them for diagnosis purpose in a cost-effective manner. In this study, we have expanded the use of specific molecular beacon probes in real-time PCR for either simultaneous detection of three Lyme spirochete species and distinguishing them using the denaturation profile analysis or detection of the presence of *A. phagocytophilum* and *B. microti* along with *B. burgdorferi* in the sample using a single assay. Use of our duplex versus a multiplex assay according to need will be efficient and less expensive assay for diagnosis of multiple tick-borne diseases.

Our optimized multiplex assay could accurately detect and quantify a single spirochete *recA* gene copy spiked in the human DNA. The presence of high concentrations of human genomic DNA (containing 10^5^ copies of ACTA1 gene) did not affect accuracy of the assay (Figure 2) as also shown by almost perfect coefficient of correlation (r^2^ = 0.999) between threshold cycle and copy number of *B. burgdorferi* DNA. In addition, an asymmetric PCR was able to detect *B. burgdorferi*, *B. afzelii* and *B. garinii* efficiently. Furthermore, it is possible to distinguish these three species using meting curve following the PCR assay (Figure 7). Using similar strategy, additional *Borrelia* species, such as emerging *B. miyamotoi*, can be identified in the future with a little tweaking of the assay.

The best time to develop an efficient diagnostic assay is when infections by a particular organism start emerging among human or animal populations, environment or in the vectors. This ensures that a well-standardized and efficient diagnostic test is available when significant population starts getting affected by the emerging pathogen. The infections of tick populations by two tick-borne pathogens, *A. phagocytophilum* and *Babesia* species have been increasing in both Europe and the United States, and the cases of infections by these emerging pathogens are also getting reported at a higher numbers in both continents [1,2]. Indeed, coinfections with these tick-borne pathogens have started appearing in the patients, and result in more severe illnesses than those observed when the patient is infected by each pathogen individually [27,81]. Therefore, we decided to expand our real-time PCR approach to include detection of these two emerging pathogens. Optimized PCR conditions for each emerging pathogen, *B. microti* and *A. phagocytophilum* BmTPK and APH1387 gene amplicons, respectively along with the human ACTA1 amplicon (Figures 3 and 4) worked well even in quadrupex assay in which serially diluted genomic DNA of *B. burgdorferi* and human could be accurately detected in addition to BmTPK and APH1387 containing plasmid DNA (Figure 5). Similarly, a 100-fold excess of *B. microti* and *A. phagocytophilum* copy number did not affect accuracy of detection of *B. burgdorferi* (Figure 6B). Moreover, this test could detect as few as 10^3^ copies of both APH1387 and BmTPK in mixed genomic DNA presence containing an excess (upto 10^3^-fold higher or 10^6^ copy number) of *B. burgdorferi* DNA indicating the sensitivity and accuracy of the assay is maintained irrespective of the different load of the pathogens presence in the sample (Figure 6A). These results demonstrate that we can use this assay to efficiently and relatively quickly detect individual pathogens, such as *B. microti* in blood bank samples using the approach used in the Figure 3. We can also diagnose coinfections with two or three pathogens in the endemic regions for these tick-borne diseases using the quadruplex assay (Figures 5 and 6). Finally, success of our assay with *B. burgdorferi* spiked human blood indicates that we will be able to use it for diagnostic purpose in human patients (Figure 8). Although real-time PCR and other techniques have been tested for identification of Lyme spirochetes and other tick-borne pathogens individually, albeit primarily in ticks [6,78,80,82-86], this is the first comprehensive study to develop assay for sensitive detection of three tick-borne pathogens simultaneously. These assays can be easily adapted for the patient samples in the future with a little modification, if needed. Furthermore, with the recent emergence of ticks infected with deer tick virus and Powassan virus lineages in New York and Connecticut in the United States and several European countries [87-89], it will be useful to include an assay for their diagnosis. Our assay could easily be extended to include the most prevalent virus amplicon after an addition reverse transcription step. Since most real-time PCR machines are capable of detecting five fluorophore with non-overlapping spectrofluorometric spectra and we have only used four in our assay, we anticipate that achieving this goal will be relatively simple. In summary, the ability of the assay described here to detect multiple tick-borne pathogens simultaneously will be a boon for health professionals to design more effective treatment regimes for coinfections when this assay is approved for mass application.

## Conclusions

Optimized conditions and PCR parameters, including the amplicons of the conserved genes present in Lyme spirochetes, *A. phagocytophilum* and the tick-borne parasite *B. microti,* and molecular beacon probes tagged with distinct fluorophores, can detect all three pathogens in a sensitive manner. Excessive presence of any pathogen did not affect sensitivity of detection of the other pathogen present in lower dose. The real-time PCR assay described here can be used both; to detect coinfections with more than one tick-borne pathogen in the endemic regions of the USA and the European countries as well as to detect each pathogen individually with equal efficiency. Since transfusion-associated babesiosis cases and fatalities are increasing steadily, the assay can also be used for detection of *Babesia* species and *A. phagocytophilum* in blood donated to the blood banks after minor modifications. The assay will be used in the future for diagnosis of tick-borne diseases after further optimization with patient samples.

## Competing interests

None of the authors have competing personal or financial interests relevant to the publication of this manuscript. We want to disclose that S.A.E.M. is among a group of inventors who earn royalties for molecular beacon usage.

## Authors’ contribution

KC and NP designed the experiments, SAEM designed the molecular beacons and KC conducted the experiments. NP drafted the manuscript. All authors read and approved the final manuscript.
